# Sexual dysfunction after female genital mutilation/cutting: A retrospective cross-sectional study of associations with organ-specific genital anatomy, trauma history, and psychiatric comorbidity

**DOI:** 10.1371/journal.pone.0356761

**Published:** 2026-09-17

**Authors:** Emily Lock, Veronica Ades

**Affiliations:** 1 Department of Obstetrics and Gynecology, New York University Grossman School of Medicine, New York, New York, United States of America; 2 Department of Obstetrics and Gynecology, Jacobi Medical Center, Bronx, New York, United States of America; Federal Medical Centre Birnin Kudu, NIGERIA

## Abstract

Sexual dysfunction after female genital mutilation/cutting (FGM/C) is poorly characterized, limiting targeted clinical intervention. In a concentrated FGM/C referral population, we aimed to characterize patterns of sexual dysfunction and evaluate associations with organ-specific genital anatomy, trauma history, and psychiatric comorbidities among women with FGM/C. We conducted a retrospective cross-sectional study of adults with FGM/C evaluated between 2014 and 2025 in an obstetrics and gynecology clinic specializing in sexual trauma. Using standardized abstraction tools, we obtained sexual symptoms, genital examination findings, demographics, and trauma-related and psychiatric variables from the electronic medical record. Associations between these variables and three primary outcomes (loss of libido, loss of sexual pleasure, dyspareunia) were assessed using univariate logistic regression. A composite arousal–pleasure dysfunction outcome was evaluated in secondary analyses adjusted for age and organ-specific genital anatomy. Among 135 women with FGM/C, approximately two-thirds reported sexual dysfunction, most commonly dyspareunia (45.9%), loss of sexual pleasure (28.9%), and loss of libido (25.9%). Absence or attenuation of the clitoris, labia minora, or labia majora was not associated with these outcomes. In contrast, depression (odds ratio [OR] 2.80), post-traumatic stress disorder (PTSD) (OR 3.86), and intact memory of the FGM/C event (OR 2.82) were associated with loss of libido. Depression was also associated with loss of sexual pleasure (OR 4.67). After adjustment for age and genital anatomy, depression (adjusted OR 3.87) and PTSD (adjusted OR 3.67) remained associated with composite arousal-pleasure dysfunction. In this well-documented cohort with FGM/C, sexual dysfunction was more closely associated with trauma-related and psychiatric factors than with genital tissue loss. These findings support prioritizing mental health and trauma-focused interventions for sexual health after FGM/C and underscore the need for prospective multicenter studies.

## Introduction

Female genital mutilation/cutting (FGM/C), the non-medically indicated injury or removal of genital organs, has been associated with obstetric, urologic, and gynecologic complications [1]. Growing evidence also supports an association between FGM/C and sexual dysfunction, including dyspareunia, loss of libido, and reduced arousal and sexual satisfactio. [2–5]. Qualitative studies indicate that many women with FGM/C attribute diminished sexual desire to clitoral excision, and that some communities justify the practice as a means of suppressing female sexuality and perceived “promiscuity.” [6]. It remains unclear, however, whether anatomic alteration of genital structures is the principal driver of sexual dysfunction, or whether trauma-related factors and psychiatric comorbidities contribute equally or more substantially.

Understanding these mechanisms is paramount given the global scale of FGM/C, which affects over 230 million women and girls, predominantly in countries across Africa, the Middle East, and Southeast Asia [7]. The World Health Organization (WHO) classifies FGM/C by extent and configuration of tissue removal, ranging from partial or total removal of the external clitoral structures and/or labia (types I-II), to infibulation (type III), to all other harmful non-medical procedures on genitals (e.g., pricking, cauterization) (type IV) [8]. This spectrum of procedures results in considerable variation in anatomical outcomes, yet the sexual sequelae of specific anatomical variants remain poorly defined. Most quantitative studies assess FGM/C using WHO types rather than organ-specific anatomy, and findings are mixed: some describe a dose-response relationship between more extensive tissue damage and sexual dysfunction, [9–11] while others report an absence of this association [12]. These discrepancies likely reflect anatomic heterogeneity within each WHO type and highlight the need for investigations that clearly define presence, partial presence, or absence of individual genital structures.

Traumatic and psychological factors may further complicate the relationship between anatomy and sexual dysfunction. Conditioned fear of genital contact, shame, altered body image, and flashbacks likely contribute to sexual distress, however empirical data directly linking these mechanisms to sexual outcomes remain limited [13,14]. Women with FGM/C demonstrate high rates of depression and post-traumatic stress disorder (PTSD), conditions that are independently associated with sexual dysfunction in general populations [15–22]. Despite this complex psychosocial context, surgical interventions such as clitoral reconstruction are increasingly offered to restore genital appearance and sexual function, although safety and efficacy data are still evolving [23–25]. Accordingly, there is persistent uncertainty about whether loss of visible genital tissue is the primary modifiable determinant of sexual outcomes, or whether psychological mechanisms are more salient targets for intervention.

This retrospective cross-sectional study aimed to characterize sexual dysfunction among women with prior FGM/C and to identify factors associated with the most commonly reported sexual complaints. Specifically, we examined patterns of sexual symptoms in relation to organ-specific genital anatomy, trauma history, and psychiatric comorbidities, with the goal of identifying clinically relevant correlates. We hypothesized that among women with FGM/C, sexual dysfunction would relate more strongly to trauma-related and psychiatric factors than to organ-specific external genital tissue loss.

## Methods

### Study design and setting

We conducted a retrospective cross-sectional chart review of women with FGM/C receiving obstetric and gynecologic (OB-GYN) care at the EMPOWER Center between January 2014 and November 2025. The EMPOWER Center exists within a safety-net health system, New York City Health + Hospitals Corporation (NYC HHC), that provides integrated, trauma-informed OB-GYN and psychiatric services to women with a history of sexual trauma, including those undergoing medical evaluation for asylum. Patients are referred from community organizations, legal and social services, and the Program for Survivors of Torture (PSOT), a multidisciplinary network of medical, psychiatric, social, and legal providers. Intake OB-GYN visits include standardized documentation of trauma and psychiatric history and detailed genital and pelvic examination.

This study was approved by the New York University Langone Health Institutional Review Board (ID i25-01552). A waiver of informed consent was granted given the research involved minimal risk and was limited to retrospective review of existing clinical data. This study adheres to the STROBE guidelines, with the completed checklist provided in Supporting Information (S2 File).

### Participants

Eligible participants were adult women presenting to the EMPOWER Center during the study period. Inclusion criteria were: (1) age ≥ 18 years at EMPOWER OB-GYN intake visit; (2) documentation of genital examination performed by EMPOWER OB-GYN provider consistent with FGM/C; and (3) documentation of partnered sexual activity after FGM/C during EMPOWER intake. Participants were excluded if the genital exam was missing or insufficient to confirm FGM/C, or if post-FGM/C sexual activity could not be determined. All patients presenting during the study period that met these criteria were included, and no random selection was performed.

### Data sources and abstraction

Data were retrospectively obtained December 16–28 2025 from the NYC HHC electronic medical record (EMR) and entered into a standardized database in REDCap, an electronic data capture tool hosted at New York University. Demographic, anatomic, trauma-related, psychiatric, and sexual dysfunction variables were extracted from intake OB-GYN notes and, when available, linked psychiatric documentation. Authors had access to personal identifiable information during data collection, but all data were de-identified prior to data analysis. Data abstraction was performed by a clinical investigator (EL) using predefined definitions and coding rules. Ambiguous cases were reviewed with the senior author (VA) to reach consensus.

### Variable definitions

External genital anatomy at the index examination was assessed for the glans clitoris, labia minora, and labia majora. For each structure, the clinician routinely recorded the anatomic status as intact, partially excised/attenuated, or absent. Intact indicated normal appearing tissue without evidence of excision or trauma, partially excised/attenuated indicated visibly reduced or scarred tissue that was not completely absent, and absent indicated no externally visible corresponding tissue. For analysis, partially excised/attenuated and absent were combined into a single absent/attenuated category to avoid very small cell counts limiting model stability, while still addressing our primary interest in comparing visible tissue distortion with intact anatomy.

Trauma-related, binary variables extracted from standardized and narrative documentation included: intact memory of the FGM/C event, lifetime sexual abuse, and lifetime intimate partner violence (IPV). Lifetime sexual abuse and IPV were based on clinician-recorded patient disclosure, documented in the dedicated violence history section of the structured Ob/Gyn intake note.

Psychiatric diagnoses were obtained for participants with documented evaluation or treatment by a licensed mental health professional within the EMPOWER Center, PSOT, or elsewhere in NYC HHC. Among psychiatric diagnoses, depression and PTSD were the most frequently documented conditions and have well-established associations with sexual function in the literature. We therefore focused our regression analyses on these two diagnoses as primary psychiatric predictors to preserve power. Depression was defined as a DSM-5 diagnosis of major depressive disorder or bipolar disorder with a depressive episode. This clinically evaluated subset may not capture undiagnosed or external psychiatric diagnoses without documentation.

Since no validated sexual function instrument was routinely administered in this clinical setting, sexual symptoms were abstracted from narrative intake OB-GYN notes. Symptoms were coded as present if explicitly documented by the clinician, while absence of documentation was treated as absence of the symptom. The three most commonly reported outcomes (loss of libido, loss of sexual pleasure, dyspareunia) were prespecified as primary sexual dysfunction outcomes for analysis to concentrate inference on the most salient complaints. Loss of libido and loss of sexual pleasure reflect terminology used in clinical documentation. For participants who underwent FGM/C in early childhood, these terms may more accurately reflect a perceived absence or deficit rather than a change from a prior experienced baseline. Given the qualitative nature of symptom ascertainment, we also constructed a composite arousal–pleasure dysfunction outcome as part of exploratory, secondary analyses, defined as presence of any of the following: anorgasmia, loss of libido, loss of sexual pleasure, or loss of genital sensation. Dyspareunia was excluded from this composite variable to avoid conflating pain-related and desire/arousal domains.

### Statistical analysis

Descriptive statistics summarized demographic, anatomic, trauma-related, psychiatric, and sexual dysfunction variables. Continuous variables were summarized using mean and standard deviation (SD), and categorical variables using frequencies and percentages.

For the three primary sexual dysfunction outcomes, we used univariate logistic regression to evaluate associations with demographic, anatomic, trauma-related, and psychiatric predictors. Psychiatric predictors (depression, PTSD) were analyzed only within the subset with documented psychiatric evaluation. Logistic regression models were fitted using maximum likelihood estimation. Results were reported as odds ratios (ORs) with 95% confidence intervals (CIs).

Given the non-standardized, qualitative assessment of sexual symptoms, we conducted prespecified secondary analysis using the composite arousal–pleasure dysfunction outcome. For these analyses, we focused on trauma-related and psychiatric variables with strong clinical plausibility or prior evidence of association with sexual function: depression, PTSD, intact memory of FGM/C event, and lifetime sexual abuse. We fit separate logistic regression models for each predictor, examining its association with composite arousal–pleasure dysfunction while adjusting a priori for age at intake and organ-specific genital anatomy. Adjusted odds ratios (aORs) and 95% CIs were reported.

Analyses were performed using R statistical software (version 4.3.2; R Core Team, 2023). All hypothesis tests were two-sided with ɑ = 0.05. We did not perform a formal sample size calculation because the cohort size was determined by the available population during the study period. Aside from psychiatric variables, missing data were limited to religion, country of origin, and age at FGM/C, as reported in Table 1, Table 2, Table 3, Table 4.

**Table 1:** Demographic characteristics.

Characteristic	n	% / Mean (SD)
Mean Age at Intake (yrs)	135	36.1 (9.2)
Age FGM/C was Performed (yrs)	131	9.0 (7.3)
Country of Origin		
Guinea	41	30.4
Burkina Faso	24	17.8
Nigeria	21	15.6
Ghana	12	8.9
Ivory Coast	9	6.7
Mali	5	3.7
Sierra Leone	4	3.0
Chad	3	2.2
Gambia	3	2.2
Other African Country	11	8.1
Not specified	2	1.5
Religion		
Muslim	61	45.2
Christian	15	11.1
Catholic	8	5.9
Other	9	6.7
Not specified	42	31.1
Primary Language		
French	67	49.6
English	59	43.7
Other	9	6.7
Source of Referral		
Lawyer/Legal Assistance Group	58	43.0
Program for Survivors of Torture	54	40.0
Other Social Service Organization/NGO	15	11.1
Healthcare Provider	6	4.4
Self	2	1.5
Reason for Intake Visit ^a^		
FGM/C Exam or Affidavit	122	90.4
Specific Symptom	35	25.9
Routine Exam	31	23.0
Affidavit for other reason	6	4.4
Family Planning	2	1.5
Pregnancy	2	1.5
FGM/C Type		
I	16	11.9
II	104	77.0
III	10	7.4
IV	1	0.7
Not specified	4	3.0

^a^Reasons for intake visit are not mutually exclusive.

**Table 2:** Genital Anatomy, Trauma/Psychiatric Comorbidity, and Sexual Dysfunction.

Variable/Levels	n	%
**Anatomy**		
Clitoris Anatomy		
Intact	27	20.0
Attenuated	23	17.0
Absent	85	63.0
Labia Minora Anatomy		
Intact	21	15.6
Attenuated	44	32.6
Absent	70	51.9
Labia Majora Anatomy		
Intact	69	51.1
Attenuated	14	10.4
Absent	52	38.5
**Trauma/Psychiatric Comorbidity**		
Traumatic Events		
Memory of FGM/C Event	98	72.6
Sexual Abuse	46	34.1
Intimate Partner Violence	56	41.5
Psychiatric Diagnosis ^a^		
Depressive Disorder	32	36.4
PTSD	20	22.7
Other Diagnosis	7	8.0
**Sexual Dysfunction** ^b^		
Any dysfunction	89	65.9
Dyspareunia	62	45.9
Loss of sexual pleasure	39	28.9
Loss of libido	35	25.9
Anorgasmia	15	11.1
Loss of feeling/sensation	13	9.6
Post-coital bleeding	6	4.4
Inability to penetrate	4	3.0

PTSD = post-traumatic stress disorder. ^a^ Includes patients who received psychiatric evaluation (n = 88/135). ^b^ Patient-reported sexual dysfunction symptoms are not mutually exclusive.

**Table 3:** Univariate Analysis of Anatomic, Trauma-Related, and Psychiatric Correlates of Sexual Dysfunction.

	Loss of Libido			Loss of Pleasure			Dyspareunia		
Variable/Level	n (%)	OR (95% CI)	p	n (%)	OR (95% CI)	p	n (%)	OR (95% CI)	p
**Anatomy**									
Clitoris									
Abs/Attn.	26 (24.1)	0.63 (0.26–1.63)	0.329	31 (28.7)	0.96 (0.39–2.52)	0.924	52 (48.1)	1.58 (0.67–3.87)	0.302
Intact	9 (33.3)	—		8 (29.6)	—		10 (37.0)	—	
Labia Minora									
Abs/Attn.	32 (28.1)	2.34 (0.73–10.48)	0.196	36 (31.6)	2.77 (0.87–12.35)	0.120	56 (49.1)	2.41 (0.91–7.17)	0.089
Intact	3 (14.3)	—		3 (14.3)	—		6 (28.6)	—	
Labia Majora									
Abs/Attn.	14 (21.2)	0.62 (0.28–1.34)	0.224	19 (28.8)	0.99 (0.47–2.09)	0.980	35 (53.0)	1.76 (0.89–3.50)	0.106
Intact	21 (30.4)	—		20 (29.0)	—		27 (39.1)	—	
**Trauma-related/Psychiatric**									
Sexual Abuse									
Yes	15 (32.6)	1.67 (0.75–3.69)	0.205	12 (26.1)	0.81 (0.36–1.78)	0.606	27 (58.7)	2.19 (1.07–4.58)	0.034*
No	20 (22.5)	—		27 (30.3)	—		35 (39.2)	—	
IPV									
Yes	17 (30.4)	1.48 (0.68–3.22)	0.324	16 (28.6)	0.97 (0.45–2.07)	0.945	31 (55.4)	1.92 (0.96–3.87)	0.065
No	18 (22.8)	—		23 (29.1)	—		31 (39.2)	—	
Memory									
Yes	30 (30.6)	2.82 (1.08–8.88)	0.050*	31 (31.6)	1.68 (0.71–4.31)	0.255	49 (50.0)	1.85 (0.85–4.13)	0.125
No	5 (13.5)	—		8 (21.6)	—		13 (35.1)	—	
Depression ^a^									
Yes	13 (40.6)	2.80 (1.07–7.49)	0.037*	14 (43.8)	4.67 (1.71–13.53)	0.003*	19 (59.4)	1.81 (0.76–4.44)	0.186
No	11 (19.6)	—		8 (14.3)	—		25 (44.6)	—	
PTSD ^a^									
Yes	10 (50.0)	3.86 (1.34–11.30)	0.012*	8 (40.0)	2.57 (0.86–7.51)	0.084	12 (60.0)	1.69 (0.62–4.80)	0.312
No	14 (20.6)	—		14 (20.6)	—		32 (47.1)	—	

Abs/Attn. = Absent or attenuated; IPV = intimate partner violence. Memory = intact memory of FGM/C event. PTSD = post-traumatic stress disorder. ^a^ Includes patients who received psychiatric evaluation (n = 88/135). *p < 0.05

**Table 4:** Univariate and Multivariate Analysis of Trauma-Related and Psychiatric Predictors of Composite Arousal–Pleasure Dysfunction.

Variable/Level	n (%)	Univariate OR (95% CI)	Univariate p	Adjusted ^a^ OR (95% CI)	Adjusted p
Depression ^b^					
Yes	21 (65.6)	4.38 (1.77–11.39)	0.002*	3.87 (1.48–10.72)	0.007*
No	17 (30.4)	—		—	
PTSD ^b^					
Yes	14 (70.0)	4.28 (1.51–13.44)	0.008*	3.67 (1.21–12.36)	0.026*
No	24 (35.3)	—		—	
FGM/C Memory					
Yes	50 (51.0)	2.17 (1.00–4.93)	0.056	1.88 (0.62–6.16)	0.273
No	12 (32.4)	—		—	

IPV = intimate partner violence; PTSD = post-traumatic stress disorder. ^a^ Multivariable logistic regression model adjusted for patient age at intake and anatomic status of clitoris, labia minora, and labia majora. ^b^ Includes patients who received psychiatric evaluation (n = 88/135). *p < 0.05

## Results

Demographic and clinical characteristics of the 135 included participants (S1 Fig) are presented in Table 1. The mean age at intake was 36.1 years (SD = 9.2), and FGM/C was performed at a mean age of 9.0 years (SD = 7.3). Most women originated from West African countries, with Guinea (30.4%, n = 41), Burkina Faso (17.8%, n = 24), and Nigeria (15.6%, n = 21) the most common countries of origin. The vast majority presented for FGM/C-related asylum affidavit examination (90.4%, n = 122), although a substantial subset also sought care for specific symptoms (25.9%, n = 35) or routine gynecologic care (23.0%, n = 31). Over three-quarters had type II FGM/C (77.0%, n = 104). Among the 10 women with Type III FGM/C, 3 remained infibulated at intake, 5 had prior vaginal births resulting in deinfibulation, and 2 had no history of childbirth but were sexually active with examination findings consistent with deinfibulation, presumed secondary to intercourse.

Genital anatomy findings, trauma and psychiatric history, and sexual dysfunction symptoms are summarized in Table 2. The glans clitoris was absent in nearly two-thirds of participants (63.0%, n = 85), with an additional 17.0% (n = 23) demonstrating attenuated clitoral tissue. Most women had attenuated (32.6%, n = 44) or absent (51.9%, n = 70) labia minora, while the majority had intact labia majora (51.1%, n = 69).

Approximately two-thirds of women (65.9%, n = 89) reported at least one sexual dysfunction symptom. The most frequently reported symptoms were dyspareunia (45.9%, n = 62), loss of sexual pleasure (28.9%, n = 39), and loss of libido (25.9%, n = 35). Less commonly reported symptoms included anorgasmia (11.1%, n = 15), loss of genital sensation (9.6%, n = 13), post-coital bleeding (4.4%, n = 6), and inability to achieve penetration (3.0%, n = 4). Trauma exposure was common, with over 72.6% (n = 98) of participants reporting memory of the FGM/C event, 41.5% (n = 56) reporting IPV, and 34.1% (n = 46) reporting lifetime sexual abuse. Among the subset of patients with documented psychiatric evaluation (n = 88), 36.4% (n = 32) had a depressive disorder and 22.7% (n = 20) had PTSD.

Associations between anatomic, trauma-related, and psychiatric variables and the three primary sexual dysfunction outcomes are shown in Table 3. Compared with women with an intact clitoris, those with an absent or attenuated clitoris did not have significantly different odds of loss of libido, loss of sexual pleasure, or dyspareunia. Labia minora and labia majora status were also not statistically associated with loss of libido or loss of sexual pleasure. Compared to women with intact structures, dyspareunia was most frequent among women with absent/attenuated labia minora (49.1% versus 28.6%) and labia majora (53% versus 39.1%), however these differences were not statistically significant (labia minora OR 2.41, 95% CI 0.91–7.17; labia majora OR 1.76, 95% CI 0.89–3.50).

Intact memory of the FGM/C event was associated with higher odds of loss of libido (OR 2.82, 95% CI 1.08–8.88, p = 0.05) but was not clearly related to loss of sexual pleasure or dyspareunia. Lifetime sexual abuse was associated with more than twice the odds of dyspareunia (OR 2.19, 95% CI 1.07–4.58, p = 0.034), without apparent associations with loss of libido or pleasure. IPV was not significantly associated with the three primary sexual outcomes.

In the clinically evaluated psychiatric subset, depression was associated with higher odds of loss of libido (OR 2.80, 95% CI 1.07–7.49, p = 0.037) and loss of sexual pleasure (OR 4.67, 95% CI 1.71–13.53, p = 0.003). PTSD was associated with increased odds of loss of libido (OR 3.86, 95% CI 1.34–11.30, p = 0.012) and showed a positive trend with loss of sexual pleasure that did not reach statistical significance (OR 2.57, 95% CI 0.86–7.51). Dyspareunia was not significantly associated with depression or PTSD.

In exploratory analyses evaluating the composite arousal–pleasure dysfunction outcome, both depression (OR 4.38, 95% CI 1.77–11.39, p = 0.002) and PTSD (OR 4.28, 95% CI 1.51–13.44, p = 0.008) were associated with increased odds of dysfunction in univariate models (Table 4). In models adjusted for age and clitoris, labia minora, and labia majora anatomy, these associations remained significant (depression adjusted OR [aOR] 3.87, 95% CI 1.48–10.72, p = 0.007; PTSD aOR 3.67, 95% CI 1.21–12.36, p = 0.026). Intact memory of the FGM/C event and lifetime sexual abuse showed positive, non-significant trends with the composite outcome after adjustment.

## Discussion

FGM/C is highly prevalent globally with well-documented health consequences, yet the mechanisms underlying sexual dysfunction in survivors remains poorly understood. In this cross-sectional sample, most participants reported sexual dysfunction following FGM/C, underscoring the need for tailored, evidence-based treatment guidelines. This study adds to the literature by using organ-specific assessment of external genital anatomy rather than relying solely on aggregate WHO FGM/C types (I-IV), and by integrating documented psychiatric diagnoses with sexual outcomes. We found no clear associations between excision of the clitoris, labia minora, or labia majora and the most common sexual dysfunction symptoms, whereas depression, PTSD, and intact memory of the FGM/C event were associated with libido and pleasure disturbances. These findings support a focus on psychological and trauma-related mechanisms alongside anatomic considerations when evaluating sexual dysfunction in FGM/C survivors.

Several mechanisms may explain the absence of association between genital tissue loss and sexual symptoms in this cohort. Erogenous sensitivity may shift to alternative anatomic sites following FGM/C, with women with FGM/C more frequently identifying the breasts as most sensitive rather than the clitoris [26]. In addition, the clitoral body, crura, and vestibular bulbs typically remain intact after FGM/C, [27] preserving capacity for vasocongestion, arousal, and orgasm through these subcutaneous structures [28]. Local tissue and peripheral nerve injury would also not be expected to directly alter ovarian hormone production that influences sexual desire. The dissociation between anatomy and sexual function in our cohort is consistent with prior work reporting that clitoral volume after FGM/C was not directly associated with sexual satisfaction scores [28]. While larger clitoral volume correlated with higher sexual function in women without FGM/C, non-anatomic factors appear to predominate among those with FGM/C [28].

Our results align with broader literature in FGM/C-affected immigrant communities showing that psychosocial determinants, such as discrimination and social support, can overshadow FGM/C status itself in predicting well-being [29]. The observed associations between depression, PTSD, and arousal–pleasure disturbances in our clinically evaluated subset are notable given the high prevalence of these psychiatric conditions in many FGM/C survivor populations [20,30]. Prior work in Somalia has shown that sexual dysfunction can predict PTSD in populations with FGM/C, suggesting a potential bidirectional relationship in which trauma and sexual difficulties may reinforce one another [16]. While no studies have directly elucidated pathways mediating the PTSD–sexual dysfunction relationship in patients with FGM/C, dysregulation of the hypothalamic-pituitary-adrenal (HPA) axis represents a plausible mechanism. Higher cortisol has been associated with decreased sexual arousal in chronically stressed women, [31] and elevated hair cortisol has been observed among women undergoing FGM/C at younger ages and after more extensive excision [32]. These findings imply that timing and anatomic extent of FGM/C may differentially affect stress physiology and, in turn, sexual function.

We also found that intact memory of the FGM/C event was associated with reduced libido, a pattern consistent with undergoing FGM/C at an older age when autobiographical memory is more developed. While sexual outcomes have not been directly compared by memory status, vivid recollection of FGM/C has been linked to increased psychopathology [15]. Additionally, PTSD appears to be more common among women undergoing FGM/C at older ages [17]. Taken together with prior work on early-life stress and HPA axis dysregulation, [31,32] these distinct patterns raise the possibility that stress at a young age and consciously remembered trauma may influence sexual function through discrete pathways. However, the role of FGM/C memory in sexual health remains underexplored and our findings should be interpreted as preliminary and hypothesis-generating.

Clinically, our findings do not support a purely anatomy-centered response to FGM/C-related sexual concerns. At the same time, qualitative studies indicate that many women seeking clitoral reconstruction primarily attribute their sexual difficulties to loss of clitoral tissue rather than to “psychological blocks.” [13]. Accordingly, effective counseling should validate patient experiences while communicating the complex biopsychosocial determinants of sexual pleasure. Educational and psychosexual interventions that address sexual physiology and relaxation techniques have improved sexual desire for women with hypoactive sexual desire disorder, [33] and may have applications for FGM/C-affected populations. Clinician training is critical, as provider knowledge gaps and concerns about cultural misunderstanding have been cited as barriers to effective counseling for patients with FGM/C [34,35]. Given the potential risks of re-traumatization associated with genital surgery [36,37]. Psychological and multidisciplinary approaches should be considered central components of care while higher-quality evidence on surgical and integrated care models continue to develop [38,39].

These findings carry implications for both clinicians and policymakers. The WHO has recently included clitoral reconstruction among its recommended FGM/C sexual dysfunction interventions, reflecting growing demand and development of evidence for its use in select patients [40]. However, trauma-informed psychological support and psychosexual counseling should be considered core treatment components, rather than adjuncts to surgical intervention, based on our observational results. Clinician training should address biopsychosocial determinants of sexual health after FGM/C while more substantial prospective research on integrated OB-GYN and psychiatric care pathways tailored to FGM/C survivors is conducted. Clinical guidelines should ultimately be updated to reflect the primacy of psychosocial factors in FGM/C-related sexual dysfunction. Accordingly, policymakers should invest in integrated, multidisciplinary service models prioritizing psychiatric and psychosexual services alongside gynecologic care, particularly in healthcare systems serving large immigrant and asylum-seeking populations.

Several limitations warrant consideration. The cross-sectional design precludes determination of temporal relationships or causal inference among FGM/C, trauma history, psychiatric comorbidity, and sexual dysfunction. Sexual dysfunction was identified from narrative EMR documentation rather than standardized instruments, which may have led to under-identification or misclassification of symptoms that were not explicitly discussed or recorded. Additionally, we were unable to assess status of all genital structures, including the clitoral hood and prepuce, or the presence, location, and extent of scarring, which may contribute to sexual symptoms. Psychiatric diagnoses were available only for a subset of patients with formal psychiatric evaluation, raising the possibility of selection bias toward individuals with greater symptom burden or recognized mental health concerns. We were unable to adjust for all potential confounders, including relationship dynamics, medication use, menopause, childbirth experiences, and other medical conditions. Given the modest sample size, some subgroups were small, yielding wide confidence intervals and limited precision. Missing data were also present for several variables, most notably psychiatric diagnosis (n = 88/135, 35% missingness), as these data required documented evaluation by a licensed mental health professional. This psychiatric data missingness may reflect differential access to or engagement with mental health services, potentially introducing selection bias. While we focused on the three most common sexual dysfunction outcomes and hypothesis-driven comparisons (anatomical versus psychosocial factors), we did not adjust for multiple comparisons and therefore regression findings should be interpreted as exploratory. We lacked a non-FGM/C control group and associations between genital status and sexual dysfunction may be confounded by FGM/C exposure itself; however, this limitation is inherent to our study aim of disentangling anatomic, trauma-related, and psychiatric correlates specifically among those with FGM/C. Finally, the predominance of immigrant, asylum-seeking patients in a single urban safety-net health system may limit generalizability to other settings.

This study also has important strengths. Organ-level genital assessment and multivariable adjustment for age and external anatomy enabled a mechanism-focused exploration of sexual dysfunction among women with FGM/C. The integration of gynecologic and psychiatric data within a single health system, along with structured documentation of trauma history, FGM/C event details, and genital anatomy at the specialized OB-GYN clinic, provided a clinically rich dataset for observational analysis. It is notable that this detailed data could be obtained from this concentrated referral base, given that systematic documentation of FGM/C and its sequelae remain limited globally. Future pooling of data across FGM/C-specialized clinical programs would increase sample sizes and enable more precise evaluation of the psychiatric outcomes identified here.

## Conclusions

These findings challenge the assumption that genital tissue loss alone drives sexual dysfunction in FGM/C survivors and instead highlights trauma-related and psychological mechanisms as key, potentially modifiable targets. Our results support prioritizing multidisciplinary, trauma-informed approaches to sexual symptom management over exclusively anatomy-centered operative solutions with uncertain benefits and potential harms. Prospective studies using validated sexual function measures and evaluations of integrated care models are needed to develop evidence-based treatment guidelines for this large, historically understudied group.

## Supporting information

S1 Fig Flow diagram depicting patient inclusion and exclusion for the cross-sectional study. Numbers of patients assessed for eligibility, excluded, and included in the overall analytic cohort and psychiatric subset are displayed.
(DOC)

S2 File STROBE statement for cross-sectional studies. Completed STROBE checklist indicating where each item is addressed in the manuscript.
(DOCX)
