# Supplementary material for: Sexual dysfunction after female genital mutilation/cutting: A retrospective cross-sectional study of associations with organ-specific genital anatomy, trauma history, and psychiatric comorbidity
Source: PLoS One. 2026 Sep 17;21(9):e0356761. doi: 10.1371/journal.pone.0356761 (PMC13585219; doi:10.1371/journal.pone.0356761)
Supplement: Supplementary file 10002 [file pone.0356761.s004.pdf]

We thank the Academic Editor and all four reviewers for their thoughtful and constructive comments. We have carefully addressed each point below, with all changes reflected in the revised manuscript.

<u>Response to the Academic Editor</u>

Editor Comment 1: *The design of study should feature in the title*

Response: We revised the title to include the study design. The new title is:

> "Sexual dysfunction after female genital mutilation/cutting: A retrospective cross-sectional study of associations with organ-specific genital anatomy, trauma history, and psychiatric comorbidity"

Editor Comment 2: *Line 112 is not clear.*

Response: Line 112 in the original manuscript read "Authors had access to information that could identify individual participants during data collection, but not after during data analysis."

We have revised it to:

> "Authors had access to personal identifiable information during data collection, but all data were de-identified prior to data analysis." (Lines 116-117)

Editor Comment 3: *Under the limitation, highlight the small sample and the missing data*

Response: We expanded the limitations section to more explicitly address the modest sample size and extent of missing data. (Lines 358-363)

Editor Comment 4: *Add implication of findings to policymakers and healthcare professionals*

Response: We added a dedicated paragraph to the Discussion addressing policy and clinical practice implications. (Lines 333-345)

Editor Comment 5: *Shorten the conclusion*

Response: We have condensed the Conclusions section. (Lines 385-391)

<u>Response to Reviewer #1</u>

Reviewer #1 Comment: *the terms "loss of sexual pleasure" and "loss of libido" imply that such experiences were previously present. However, given that many participants underwent excision at a young age (e.g., around 9 years old), it is possible that they may not have had substantial prior experiences of sexual pleasure or libido. In this specific context, wording such as "lack of sexual pleasure" or "reduced capacity for sexual pleasure" might be more precise. This is, however, a minor point for consideration rather than a substantive critique.*

Response: We agree that the phrase "loss of" presumes a prior baseline that may not apply to participants who underwent FGM/C before sexual maturity. Because this terminology was directly abstracted from clinical documentation, we retained it to preserve fidelity to the source data. However, we address this consideration in the "Variable Definitions" subsection of Methods (Lines 151-154)

<u>Response to Reviewer #2</u>

Reviewer #2 Comment 1: *Lines 16 and 71 use slightly different combinations of terminology. I think the fact it is retrospective needs to be stated in the methods on line 71.*

Response: Wording clarified in line 74 (previously 71) to clarify retrospective nature:

Reviewer #2 Comment 2: *Line 49 mentions "partial or total removal of the clitoris...". This reference to "total removal" is anatomically inaccurate since no FGM/C removes the entire clitoris. I am confident the authors know this as they discuss (lines 270-272) clitoral features that remain intact regardless of FGM/C type later in the manuscript. Please correct the language on line 49 to acknowledge that it is a portion of external clitoral structures that are removed in some types of FGM/C. This will help both clinicians with poor clitoral anatomy knowledge and women who have heard - and are additionally traumatized by - misinformation about their entire clitoris being gone.*

Response: We agree that WHO classification language can perpetuate anatomically inaccurate misconceptions about the extent of clitoral loss. We changed "clitoris" to "external clitoral structures" (Lines 50-53)

Reviewer #2 Comment 3: *Why was the status of the hood or prepuce of the clitoris not part of the definitions for this study? Given that the status of this tissue figures prominently into FGM types, a rationale for this decision should be included.*

Response: We agree these are important structures, however the status was not reliably recorded for all patients, so we added a sentence in the Discussion to acknowledge the limitation (Lines 351-353):

Reviewer #2 Comment 4: *I understand and was happy to see this study focused on specific anatomy and not FGM Types. However, there was no mention of scars or their length which, in my experience (conducting about 40 such exams in the last 3 years), has an impact on sexual function. Did your study take the presence/absence of scar tissue into account? If not, that should be added to the limitations because scars cannot be ruled out as playing a role in the aspects of sexual function discussed in this study.*

Response: We thank the reviewer for this clinically important observation. We agree that scarring can contribute to dyspareunia/other sexual symptoms independently of tissue absence. Scar tissue was not systematically extracted as a variable in this study because clinical documentation did not consistently characterize scar presence, extent, or location as discrete items. We added text explaining this (Line 351-353)

Reviewer #2 Comment 5: *Only a small portion of the sample is listed as having Type 3. Please add - if possible - whether or not the women had been defibulated or not when their sexual function data was collected. If not known, that's okay, but state that.*

Response: We have added clarification of infibulation status at time of intake in the Results (lines 196-199).

Reviewer #2 Comment 6: *Were women considered to have IPV is they had any lifetime history and/or current IPV? Also, was a standardized tool used to screen or was it only recorded if it was present in the clinical note? Please specify.*

Response: We thank the reviewer for requesting this methodology clarification. IPV (lifetime) was coded as present if it was documented in a dedicated section of the Ob/Gyn intake note for violence history based on patient report upon physician questioning. We clarified this in the “Variable Definitions” subsection in Methods (lines 133-135)

Reviewer #2 Comment 7: *Menopause and childbirth are both associated with changes in sexual function. Although it is beyond the scope of this study to address those changes in detail, I would recommend it be acknowledged in the limitations.*

Response: We have added menopause and childbirth experiences to the list of potential influences on sexual function that we could not account for (Lines 356-358).

Reviewer #2 Comment 8: *The authors allude to the limits of clitoral reconstruction/restoration (CR) in three places: lines 65-67, 315-316 and 354-355. This is fair, however, it would be helpful to acknowledge that the WHO has recently included CR in its recommendations for women with FGM/C and that this reflects growing evidence to support its use. That should be acknowledged. In fact, the data presented here is more important than ever because the change in the WHO recommendations may mean clinicians move even more quickly to CR options - especially if they do not read this paper and use its finding to help patients make decisions about whether or not to have CR.*

Response: We thank the reviewer for drawing attention to the latest guidelines by WHO. We have revised the Discussion to reflect this information. (Lines 332-336)

<u>Response to Reviewer #3</u>

Reviewer #3 Comment 1: *LINE 49-50: Please take a look at the FGC classification again. There is a 4th type which includes all pricking or piercing scraping and cauterization etc.*

Response: We have revised the relevant sentence in the Introduction to include all four WHO-classified types. (Lines 52-53)

Reviewer #3 Comment 2: *LINE 106: I can assume from what was detailed that there was no random selection of historical cases, all cases that qualified based on the inclusion criteria were used, right?*

Response: Correct, all patients meeting inclusion criteria were included, without use of random selection. A clarifying sentence was added to the "Participants" subsection in the Methods section. (Lines 107-109)

Reviewer #3 Comment 3: *I was trying to figure out any information about the timing of the FGC/M event, and the onset of the sexual dysfunction especially dyspareunia? Are such records available please?*

Response: We thank the reviewer for this clinically relevant question. Unfortunately, the timing of sexual dysfunction onset relative to the FGM/C event was not reliably documented in clinical records. This fundamental limitation of the retrospective cross-sectional design was acknowledged in the Discussion section of the original draft (Lines 347-349)

Reviewer #3 Comment 4: *I am not sure how you were able to determine whether the mental health issues were related to FGC/M alone or could be due to post-immigration issues and uncertainties.*

Response: We thank the reviewer for this important conceptual comment. We agree that we cannot attribute psychiatric diagnoses, or their relationship to sexual dysfunction, specifically to FGM/C. Indeed, our study population is composed predominantly of immigrant and asylum-seeking women, and the psychological sequelae of forced migration, acculturation stress, legal uncertainty, and isolation may independently contribute to both psychiatric diagnoses and sexual dysfunction. Regardless of causality, mental health disorders, which do have a high prevalence in this population, remain a significant correlate with sexual dysfunction that we believe warrants attention.

Response to Reviewer #4

Reviewer #4 Comment: *"The only thing missing in the introduction is some literature search or future assessment of how these data can be combined with other studies and datasets to further evaluate the psychiatric outcomes reported here."*

Response: We thank the reviewer for this suggestion. While we agree that situation this study within a broader data integration framework is valuable, we felt this was most appropriately addressed in the Discussion rather than the Introduction. In addition to our analysis of (Lines 293-317), we also added the following sentence to emphasize the importance of expanding to larger datasets:

"Future pooling of data across FGM/C-specialized clinical programs would increase sample sizes and enable more precise evaluation of the psychiatric outcomes identified here." (Lines 380-382)

We believe these revisions addressing all points raised by the Academic Editor and reviewers substantially strengthen the manuscript. We appreciate and look forward to the editorial team's further consideration of this work.
