# Supplementary material for: Sexual dysfunction after female genital mutilation/cutting: A retrospective cross-sectional study of associations with organ-specific genital anatomy, trauma history, and psychiatric comorbidity
Source: PLoS One. 2026 Sep 17;21(9):e0356761. doi: 10.1371/journal.pone.0356761 (PMC13585219; doi:10.1371/journal.pone.0356761)
Supplement: Supplementary file 2 [file pone.0356761.s001.doc]

**FGM/C Patients**

*(n = 141)*

Excluded *(n = 6)*

< 18 years old *(n = 2)*

Declined pelvic/genital exam *(n = 2)*

Denied sexual activity *(n = 2)*

**Overall Analytic Cohort**

*(n = 135)*


**Psychiatric Subset**

*(n = 88)*

Excluded *(n = 47)*

No qualifying psychiatric evaluation

**Supplementary Figure 1.** Flow diagram depicting patient inclusion and exclusion for the cross-sectional study. Numbers of patients assessed for eligibility, excluded, and included in the overall analytic cohort and psychiatric subset are displayed.
