# Supplementary material for: Sexual dysfunction after female genital mutilation/cutting: A retrospective cross-sectional study of associations with organ-specific genital anatomy, trauma history, and psychiatric comorbidity
Source: PLoS One. 2026 Sep 17;21(9):e0356761. doi: 10.1371/journal.pone.0356761 (PMC13585219; doi:10.1371/journal.pone.0356761)
Supplement: Peer review history

# Peer review: Decision Letter 0

**DOI:** 10.1371/journal.pone.0356761.r001

**Authors:** Adewale Ashimi

5 Jul 2026

PONE-D-26-15261

Sexual dysfunction after female genital mutilation/cutting: Associations with organ-specific genital anatomy, trauma history, and psychiatric comorbidity

PLOS One

Dear Dr. Ades,

Thank you for submitting your manuscript to PLOS ONE. After careful consideration, we feel that it has merit but does not fully meet PLOS ONE’s publication criteria as it currently stands. Therefore, we invite you to submit a revised version of the manuscript that addresses the points raised during the review process

1. The design of study should feature in the title

2.Line 112 is not clear.

3. Under the limitation, highlight the small sample and the missing data

4. Add implication of findings to policymakers and healthcare professionals.

5. Shorten the conclusion

Please submit your revised manuscript by Aug 19 2026 11:59PM. If you will need more time than this to complete your revisions, please reply to this message or contact the journal office at plosone@plos.org. When you're ready to submit your revision, log on to https://www.editorialmanager.com/pone/ and select the 'Submissions Needing Revision' folder to locate your manuscript file.

Please include the following items when submitting your revised manuscript:

- A letter that responds to each point raised by the academic editor and reviewer(s). You should upload this letter as a separate file labeled 'Response to Reviewers'.
- A marked-up copy of your manuscript that highlights changes made to the original version. You should upload this as a separate file labeled 'Revised Manuscript with Track Changes'.
- An unmarked version of your revised paper without tracked changes. You should upload this as a separate file labeled 'Manuscript'.

If you would like to make changes to your financial disclosure, please include your updated statement in your cover letter. Guidelines for resubmitting your figure files are available below the reviewer comments at the end of this letter.

If applicable, we recommend that you deposit your laboratory protocols in protocols.io to enhance the reproducibility of your results. Protocols.io assigns your protocol its own identifier (DOI) so that it can be cited independently in the future. For instructions see: https://journals.plos.org/plosone/s/submission-guidelines#loc-laboratory-protocols. Additionally, PLOS ONE offers an option for publishing peer-reviewed Lab Protocol articles, which describe protocols hosted on protocols.io. Read more information on sharing protocols at https://plos.org/protocols?utm_medium=editorial-email&utm_source=authorletters&utm_campaign=protocols.

As the corresponding author, your ORCID iD is verified in the submission system and will appear in the published article. PLOS supports the use of ORCID, and we encourage all coauthors to register for an ORCID iD and use it as well. Please encourage your coauthors to verify their ORCID iD within the submission system before final acceptance, as unverified ORCID iDs will not appear in the published article. Only the individual author can complete the verification step; PLOS staff cannot verify ORCID iDs on behalf of authors.

We look forward to receiving your revised manuscript.

Kind regards,

Adewale Olufemi Ashimi, MBChB, MPH, PhD, FWACS

Academic Editor

PLOS One

Journal Requirements:

When submitting your revision, we need you to address these additional requirements.

1. Please ensure that your manuscript meets PLOS ONE's style requirements, including those for file naming. The PLOS ONE style templates can be found at

https://journals.plos.org/plosone/s/file?id=wjVg/PLOSOne_formatting_sample_main_body.pdf and

https://journals.plos.org/plosone/s/file?id=ba62/PLOSOne_formatting_sample_title_authors_affiliations.pdf

2. We note that you have indicated that there are restrictions to data sharing for this study. PLOS only allows data to be available upon request if there are legal or ethical restrictions on sharing data publicly. For more information on unacceptable data access restrictions, please see http://journals.plos.org/plosone/s/data-availability#loc-unacceptable-data-access-restrictions.

Before we proceed with your manuscript, please address the following prompts:

a) If there are ethical or legal restrictions on sharing a de-identified data set, please explain them in detail (e.g., data contain potentially identifying or sensitive patient information, data are owned by a third-party organization, etc.) and who has imposed them (e.g., a Research Ethics Committee or Institutional Review Board, etc.). Please also provide contact information for a data access committee, ethics committee, or other institutional body to which data requests may be sent.

b) If there are no restrictions, please upload the minimal anonymized data set necessary to replicate your study findings to a stable, public repository and provide us with the relevant URLs, DOIs, or accession numbers. For a list of recommended repositories, please see

https://journals.plos.org/plosone/s/recommended-repositories. You also have the option of uploading the data as Supporting Information files, but we would recommend depositing data directly to a data repository if possible.

We will update your Data Availability statement on your behalf to reflect the information you provide

3. If the reviewer comments include a recommendation to cite specific previously published works, please review and evaluate these publications to determine whether they are relevant and should be cited. There is no requirement to cite these works unless the editor has indicated otherwise.

Please review your reference list to ensure that it is complete and correct. If you have cited papers that have been retracted, please include the rationale for doing so in the manuscript text, or remove these references and replace them with relevant current references. Any changes to the reference list should be mentioned in the rebuttal letter that accompanies your revised manuscript. If you need to cite a retracted article, indicate the article’s retracted status in the References list and also include a citation and full reference for the retraction notice.

[Note: HTML markup is below. Please do not edit.]

Reviewers' comments:

Reviewer's Responses to Questions

**Comments to the Author**

1. Is the manuscript technically sound, and do the data support the conclusions?

Reviewer #1: Yes

Reviewer #2: Yes

Reviewer #3: Yes

Reviewer #4: Yes

**********

2. Has the statistical analysis been performed appropriately and rigorously?

Reviewer #1: I Don't Know

Reviewer #2: I Don't Know

Reviewer #3: Yes

Reviewer #4: Yes

**********

3. Have the authors made all data underlying the findings in their manuscript fully available?

The PLOS Data policy

Reviewer #1: Yes

Reviewer #2: Yes

Reviewer #3: Yes

Reviewer #4: Yes

**********

4. Is the manuscript presented in an intelligible fashion and written in standard English?

Reviewer #1: Yes

Reviewer #2: Yes

Reviewer #3: Yes

Reviewer #4: Yes

**********

Reviewer #1: This manuscript makes an important contribution to the literature on the sexual health impacts of FGM/C. In particular, it usefully acknowledges sexuality as a biopsychosocial phenomenon, with attention not only to anatomical factors but also to psychological dimensions. Even though the study is exploratory, it already offers valuable insights that can inform future research in this area.

The authors appropriately acknowledge the study’s limitations, which are understandable given the sensitivity of the topic and the constraints of the available data. These limitations do not detract from the article’s key strengths. The manuscript presents plausible and well-argued hypothetical explanations for its findings, which are both convincing and generative for further investigation. While I am not a specialist in quantitative methods, the analyses appear sound and adequately support the conclusions. The writing is clear, engaging, and accessible.

One minor comment, more in the form of a conceptual reflection: the terms “loss of sexual pleasure” and “loss of libido” imply that such experiences were previously present. However, given that many participants underwent excision at a young age (e.g., around 9 years old), it is possible that they may not have had substantial prior experiences of sexual pleasure or libido. In this specific context, wording such as “lack of sexual pleasure” or “reduced capacity for sexual pleasure” might be more precise. This is, however, a minor point for consideration rather than a substantive critique.

Thank you for this important contribution to the literature.

Reviewer #2: This was an expertly written manuscript that adds to our understanding of this population's long term sequalae from FGM/C. I found the data immediately to my own clinical work so thank you. A few comments below for consideration:

Lines 16 and 71 use slightly different combinations of terminology. I think the fact it is retrospective needs to be stated in the methods on line 71.

Line 49 mentions "partial or total removal of the clitoris...". This reference to "total removal" is anatomically inaccurate since no FGM/C removes the entire clitoris. I am confident the authors know this as they discuss (lines 270-272) clitoral features that remain intact regardless of FGM/C type later in the manuscript. Please correct the language on line 49 to acknowledge that it is a portion of external clitoral structures that are removed in some types of FGM/C. This will help both clinicians with poor clitoral anatomy knowledge and women who have heard - and are additionally traumatized by - misinformation about their entire clitoris being gone.

Re: Variable definitions. Why was the status of the hood or prepuce of the clitoris not part of the definitions for this study? Given that the status of this tissue figures prominently into FGM types, a rationale for this decision should be included.

Re: FGM Types. I understand and was happy to see this study focused on specific anatomy and not FGM Types. However, there was no mention of scars or their length which, in my experience (conducting about 40 such exams in the last 3 years), has an impact on sexual function. Did your study take the presence/absence of scar tissue into account? If not, that should be added to the limitations because scars cannot be ruled out as playing a role in the aspects of sexual function discussed in this study.

Re: FGM Type 3. Only a small portion of the sample is listed as having Type 3. Please add - if possible - whether or not the women had been defibulated or not when their sexual function data was collected. If not known, that's okay, but state that.

Re: IPV. Were women considered to have IPV is they had any lifetime history and/or current IPV? Also, was a standardized tool used to screen or was it only recorded if it was present in the clinical note? Please specify.

Menopause and childbirth are both associated with changes in sexual function. Although it is beyond the scope of this study to address those changes in detail, I would recommend it be acknowledged in the limitations.

The authors allude to the limits of clitoral reconstruction/restoration (CR) in three places: lines 65-67, 315-316 and 354-355. This is fair, however, it would be helpful to acknowledge that the WHO has recently included CR in its recommendations for women with FGM/C and that this reflects growing evidence to support its use. That should be acknowledged. In fact, the data presented here is more important than ever because the change in the WHO recommendations may mean clinicians move even more quickly to CR options - especially if they do not read this paper and use its finding to help patients make decisions about whether or not to have CR.

See https://www.who.int/publications/i/item/9789240107281#:~:text=The%20process%20of%20updating%20and%20revising%20this%20guideline,%28PTSD%29%3B%20and%20surgical%20and%20non-surgical%20sexual%20health%20interventions.

Again, many thanks for a very clinically useful and technically expert manuscript.

Reviewer #3: REVIEW OF SEXUAL DYSFUNCTION AFTER FEMALE GENITAL CUTTING

INTRODUCTION

LINE 49-50: Please take a look at the FGC classification again. There is a 4th type which includes all pricking or piercing scraping and cauterization etc.

DATA SOURCES

LINE 106: I can assume from what was detailed that there was no random selection of historical cases, all cases that qualified based on the inclusion criteria were used, right?

I was trying to figure out any information about the timing of the FGC/M event, and the onset of the sexual dysfunction especially dyspareunia? Are such records available please?

RESULTS

I am not sure how you were able to determine whether the mental health issues were related to FGC/M alone or could be due to post-immigration issues and uncertainties.

Reviewer #4: This is unique and powerful work that the authors have contributed to women's health and society at large. The hypothesis itself, the analysis that follows and the population studied is presented in a fashion that is easy to understand while maintaining existing literature in mind. The only thing missing in the introduction is some literature search or future assessment of how these data can be combined with other studies and datasets to further evaluate the psychiatric outcomes reported here. Otherwise, this is a must read!

**********

what does this mean?). If published, this will include your full peer review and any attached files.

If you choose “no”, your identity will remain anonymous but your review may still be made public.

**Do you want your identity to be public for this peer review?**  For information about this choice, including consent withdrawal, please see our Privacy Policy

Reviewer #1: **Yes:**  Hannelore Van Bavel

Reviewer #2: No

Reviewer #3: **Yes:**  Seth Amponsah-Tabi

Reviewer #4: **Yes:**  Maheen Mausoof Adamson, PhD, MHL

**********

[NOTE: If reviewer comments were submitted as an attachment file, they will be attached to this email and accessible via the submission site. Please log into your account, locate the manuscript record, and check for the action link "View Attachments". If this link does not appear, there are no attachment files.]

To ensure your figures meet our technical requirements, please review our figure guidelines: https://journals.plos.org/plosone/s/figures

You may also use PLOS’s free figure tool, NAAS, to help you prepare publication quality figures: https://journals.plos.org/plosone/s/figures#loc-tools-for-figure-preparation.

NAAS will assess whether your figures meet our technical requirements by comparing each figure against our figure specifications.

# Author comment: Author response to Decision Letter 1

**DOI:** 10.1371/journal.pone.0356761.r002

6 Aug 2026

We thank the Academic Editor and all four reviewers for their thoughtful and constructive comments. We have carefully addressed each point below, with all changes reflected in the revised manuscript.

Response to the Academic Editor

Editor Comment 1: The design of study should feature in the title

Response: We revised the title to include the study design. The new title is:

“Sexual dysfunction after female genital mutilation/cutting: A retrospective cross-sectional study of associations with organ-specific genital anatomy, trauma history, and psychiatric comorbidity”

Editor Comment 2: Line 112 is not clear.

Response: Line 112 in the original manuscript read “Authors had access to information that could identify individual participants during data collection, but not after during data analysis.”

We have revised it to:

“Authors had access to personal identifiable information during data collection, but all data were de-identified prior to data analysis.” (Lines 116-117)

Editor Comment 3: Under the limitation, highlight the small sample and the missing data

Response: We expanded the limitations section to more explicitly address the modest sample size and extent of missing data. (Lines 358-363)

Editor Comment 4: Add implication of findings to policymakers and healthcare professionals

Response: We added a dedicated paragraph to the Discussion addressing policy and clinical practice implications. (Lines 333-345)

Editor Comment 5: Shorten the conclusion

Response: We have condensed the Conclusions section. (Lines 385-391)

Response to Reviewer #1

Reviewer #1 Comment: the terms “loss of sexual pleasure” and “loss of libido” imply that such experiences were previously present. However, given that many participants underwent excision at a young age (e.g., around 9 years old), it is possible that they may not have had substantial prior experiences of sexual pleasure or libido. In this specific context, wording such as “lack of sexual pleasure” or “reduced capacity for sexual pleasure” might be more precise. This is, however, a minor point for consideration rather than a substantive critique.

Response: We agree that the phrase “loss of” presumes a prior baseline that may not apply to participants who underwent FGM/C before sexual maturity. Because this terminology was directly abstracted from clinical documentation, we retained it to preserve fidelity to the source data. However, we address this consideration in the “Variable Definitions” subsection of Methods (Lines 151-154)

Response to Reviewer #2

Reviewer #2 Comment 1: Lines 16 and 71 use slightly different combinations of terminology. I think the fact it is retrospective needs to be stated in the methods on line 71.

Response: Wording clarified in line 74 (previously 71) to clarify retrospective nature:

Reviewer #2 Comment 2: Line 49 mentions "partial or total removal of the clitoris...". This reference to "total removal" is anatomically inaccurate since no FGM/C removes the entire clitoris. I am confident the authors know this as they discuss (lines 270-272) clitoral features that remain intact regardless of FGM/C type later in the manuscript. Please correct the language on line 49 to acknowledge that it is a portion of external clitoral structures that are removed in some types of FGM/C. This will help both clinicians with poor clitoral anatomy knowledge and women who have heard - and are additionally traumatized by - misinformation about their entire clitoris being gone.

Response: We agree that WHO classification language can perpetuate anatomically inaccurate misconceptions about the extent of clitoral loss. We changed “clitoris” to “external clitoral structures” (Lines 50-53)

Reviewer #2 Comment 3: Why was the status of the hood or prepuce of the clitoris not part of the definitions for this study? Given that the status of this tissue figures prominently into FGM types, a rationale for this decision should be included.

Response: We agree these are important structures, however the status was not reliably recorded for all patients, so we added a sentence in the Discussion to acknowledge the limitation (Lines 351-353):

Reviewer #2 Comment 4: I understand and was happy to see this study focused on specific anatomy and not FGM Types. However, there was no mention of scars or their length which, in my experience (conducting about 40 such exams in the last 3 years), has an impact on sexual function. Did your study take the presence/absence of scar tissue into account? If not, that should be added to the limitations because scars cannot be ruled out as playing a role in the aspects of sexual function discussed in this study.

Response: We thank the reviewer for this clinically important observation. We agree that scarring can contribute to dyspareunia/other sexual symptoms independently of tissue absence. Scar tissue was not systematically extracted as a variable in this study because clinical documentation did not consistently characterize scar presence, extent, or location as discrete items. We added text explaining this (Line 351-353)

Reviewer #2 Comment 5: Only a small portion of the sample is listed as having Type 3. Please add - if possible - whether or not the women had been defibulated or not when their sexual function data was collected. If not known, that's okay, but state that.

Response: We have added clarification of infibulation status at time of intake in the Results (lines 196-199).

Reviewer #2 Comment 6: Were women considered to have IPV is they had any lifetime history and/or current IPV? Also, was a standardized tool used to screen or was it only recorded if it was present in the clinical note? Please specify.

Response: We thank the reviewer for requesting this methodology clarification. IPV (lifetime) was coded as present if it was documented in a dedicated section of the Ob/Gyn intake note for violence history based on patient report upon physician questioning. We clarified this in the “Variable Definitions” subsection in Methods (lines 133-135)

Reviewer #2 Comment 7: Menopause and childbirth are both associated with changes in sexual function. Although it is beyond the scope of this study to address those changes in detail, I would recommend it be acknowledged in the limitations.

Response: We have added menopause and childbirth experiences to the list of potential influences on sexual function that we could not account for (Lines 356-358).

Reviewer #2 Comment 8: The authors allude to the limits of clitoral reconstruction/restoration (CR) in three places: lines 65-67, 315-316 and 354-355. This is fair, however, it would be helpful to acknowledge that the WHO has recently included CR in its recommendations for women with FGM/C and that this reflects growing evidence to support its use. That should be acknowledged. In fact, the data presented here is more important than ever because the change in the WHO recommendations may mean clinicians move even more quickly to CR options - especially if they do not read this paper and use its finding to help patients make decisions about whether or not to have CR.

Response: We thank the reviewer for drawing attention to the latest guidelines by WHO. We have revised the Discussion to reflect this information. (Lines 332-336)

Response to Reviewer #3

Reviewer #3 Comment 1: LINE 49-50: Please take a look at the FGC classification again. There is a 4th type which includes all pricking or piercing scraping and cauterization etc.

Response: We have revised the relevant sentence in the Introduction to include all four WHO-classified types. (Lines 52-53)

Reviewer #3 Comment 2: LINE 106: I can assume from what was detailed that there was no random selection of historical cases, all cases that qualified based on the inclusion criteria were used, right?

Response: Correct, all patients meeting inclusion criteria were included, without use of random selection. A clarifying sentence was added to the “Participants” subsection in the Methods section. (Lines 107-109)

Reviewer #3 Comment 3: I was trying to figure out any information about the timing of the FGC/M event, and the onset of the sexual dysfunction especially dyspareunia? Are such records available please?

Response: We thank the reviewer for this clinically relevant question. Unfortunately, the timing of sexual dysfunction onset relative to the FGM/C event was not reliably documented in clinical records. This fundamental limitation of the retrospective cross-sectional design was acknowledged in the Discussion section of the original draft (Lines 347-349)

Reviewer #3 Comment 4: I am not sure how you were able to determine whether the mental health issues were related to FGC/M alone or could be due to post-immigration issues and uncertainties.

Response: We thank the reviewer for this important conceptual comment. We agree that we cannot attribute psychiatric diagnoses, or their relationship to sexual dysfunction, specifically to FGM/C. Indeed, our study population is composed predominantly of immigrant and asylum-seeking women, and the psychological sequelae of forced migration, acculturation stress, legal uncertainty, and isolation may independently contribute to both psychiatric diagnoses and sexual dysfunction. Regardless of causality, mental health disorders, which do have a high prevalence in this population, remain a significant correlate with sexual dysfunction that we believe warrants attention.

Response to Reviewer #4

Reviewer #4 Comment: “The only thing missing in the introduction is some literature search or future assessment of how these data can be combined with other studies and datasets to further evaluate the psychiatric outcomes reported here.”

Response: We thank the reviewer for this suggestion. While we agree that situation this study within a broader data integration framework is valuable, we felt this was most appropriately addressed in the Discussion rather than the Introduction. In addition to our analysis of (Lines 293-317), we also added the following sentence to emphasize the importance of expanding to larger datasets:

“Future pooling of data across FGM/C-specialized clinical programs would increase sample sizes and enable more precise evaluation of the psychiatric outcomes identified here.” (Lines 380-382)

We believe these revisions addressing all points raised by the Academic Editor and reviewers substantially strengthen the manuscript. We appreciate and look forward to the editorial team’s further consideration of this work.

Attachment Submitted filename: Response to Reviewers.pdf

# Editor report: Decision Letter 1

**DOI:** 10.1371/journal.pone.0356761.r003

**Authors:** Adewale Ashimi

10 Aug 2026

Sexual dysfunction after female genital mutilation/cutting: Associations with organ-specific genital anatomy, trauma history, and psychiatric comorbidity

PONE-D-26-15261R1

Dear Prof Ades,

We’re pleased to inform you that your manuscript has been judged scientifically suitable for publication and will be formally accepted for publication once it meets all outstanding technical requirements.

Within one week, you’ll receive an e-mail detailing the required amendments. When these have been addressed, you’ll receive a formal acceptance letter and your manuscript will be scheduled for publication.

An invoice will be generated when your article is formally accepted. Please note, if your institution has a publishing partnership with PLOS and your article meets the relevant criteria, all or part of your publication costs will be covered. Please make sure your user information is up-to-date by logging into Editorial Manager at Editorial Manager® and clicking the ‘Update My Information' link at the top of the page. For questions related to billing, please contact billing support.

If your institution or institutions have a press office, please notify them about your upcoming paper to help maximize its impact. If they’ll be preparing press materials, please inform our press team as soon as possible -- no later than 48 hours after receiving the formal acceptance. Your manuscript will remain under strict press embargo until 2 pm Eastern Time on the date of publication. For more information, please contact onepress@plos.org.

Kind regards,

Adewale Olufemi Ashimi, MBChB, MPH, PhD, FWACS

Academic Editor

PLOS One

Additional Editor Comments (optional):

Reviewers' comments:

# Editor report: Acceptance letter

**DOI:** 10.1371/journal.pone.0356761.r004

**Authors:** Adewale Ashimi

PONE-D-26-15261R1

PLOS One

Dear Dr. Ades,

I'm pleased to inform you that your manuscript has been deemed suitable for publication in PLOS One. Congratulations! Your manuscript is now being handed over to our production team.

At this stage, our production department will prepare your paper for publication. This includes ensuring the following:

* All references, tables, and figures are properly cited

* All relevant supporting information is included in the manuscript submission,

* There are no issues that prevent the paper from being properly typeset

You will receive further instructions from the production team, including instructions on how to review your proof when it is ready. Please keep in mind that we are working through a large volume of accepted articles, so please give us a few days to review your paper and let you know the next and final steps.

Lastly, if your institution or institutions have a press office, please let them know about your upcoming paper now to help maximize its impact. If they'll be preparing press materials, please inform our press team within the next 48 hours. Your manuscript will remain under strict press embargo until 2 pm Eastern Time on the date of publication. For more information, please contact onepress@plos.org.

You will receive an invoice from PLOS for your publication fee after your manuscript has reached the completed accept phase. If you receive an email requesting payment before acceptance or for any other service, this may be a phishing scheme. Learn how to identify phishing emails and protect your accounts at https://explore.plos.org/phishing.

If we can help with anything else, please email us at customercare@plos.org.

Thank you for submitting your work to PLOS One and supporting open access.

Kind regards,

PLOS ONE Editorial Office Staff

on behalf of

Dr. Adewale Olufemi Ashimi

Academic Editor

PLOS One
